# Evaluation of expression of VDR-associated lncRNAs in COVID-19 patients

**DOI:** 10.1186/s12879-021-06248-8

**Published:** 2021-06-19

**Authors:** Mohammad Taheri, Lina Moallemi Rad, Bashdar Mahmud Hussen, Fwad Nicknafs, Arezou Sayad, Soudeh Ghafouri-Fard

**Affiliations:** 1 Skull Base Research Center, Loghman Hakim Hospital, Shahid Beheshti University of Medical Sciences, Tehran, Iran; 2 Department of Molecular and Cell Biology, Faculty of Basic Sciences, University of Mazandaran, Babolsar, Iran; 3 Department of Pharmacognosy, College of Pharmacy, Hawler Medical University, Erbil, Iraq; 4 Department of Medical Genetics, Shahid Beheshti University of Medical Sciences, Tehran, Iran

**Keywords:** COVID-19, Vitamin D receptor, VDR, lncRNA, SNHG6, SNHG16, Linc00511, Linc00346, CYP27B

## Abstract

**Background:**

Coronavirus disease 2019 (COVID-19) has been shown to cause serious health problems among them is the Acute Respiratory Distress syndrome (ARDS). Vitamin D receptor (VDR) signaling possibly partakes in the pathophysiology of this devastating complication.

**Methods:**

In the current project, we have appraised expression levels of *VDR*, *CYP27B1* and a number of associated lncRNAs in the circulation of COVID-19 patients versus healthy subjects using real-time PCR method.

**Results:**

Expression of *SNHG6* was considerably lower in COVID-19 patients compared with control subjects (Ratio of mean expression (RME) = 0.22, *P* value = 7.04E-05) and in both female and male COVID-19 patients compared with sex-matched unaffected individuals (RME = 0.32, P value = 0.04 and RME = 0.16, P value = 0.000679683, respectively). However, its expression was similar among ICU-hospitalized and non-ICU patients. Similarly, expression of *SNHG16* was lower in in COVID-19 patients compared with controls (RME = 0.20, *P* value = 5.94E-05) and in both female and male patients compared with sex-matched controls (RME = 0.32, P value = 0.04 and RME = 0.14, P value = 0.000496435, respectively) with no significant difference among ICU-hospitalized and non-ICU hospitalized patients. Expression of *VDR* was lower in COVID-19 patients compared with controls (RME = 0.42, *P* value = 0.04) and in male patients compared with male controls (RME = 0.27, P value = 0.02). Yet, expression of *VDR* was statistically similar between female subgroups and between ICU-hospitalized and non-ICU hospitalized patients. Expression levels *CYP27B*, *Linc00511* and *Linc00346* were similar among COVID-19 patients and healthy subjects or between their subgroups. Significant correlations have been detected between expression levels of *VDR*, *CYP27B* and *SNHG6*, *SNHG16*, *Linc00511* and *Linc00346* lncRNAs both among COVID-19 patients and among healthy controls with the most significant ones being *SNHG6* and *SNHG16* (r = 0.74, *P* value = 3.26e-17 and r = 0.81, *P* = 1.54e-22, respectively).

**Conclusion:**

Combination of transcript levels of *VDR*, *CYP27B* and *SNHG6*, *SNHG16*, *Linc00511* and *Linc00346* could differentiate patients from controls with AUC = 0.76, sensitivity = 0.62 and specificity = 0.81. The current data potentiate *SNHG6*, *SNHG16* and *VDR* as possible contributors in COVID-19 infection but not in the severity of ARDS.

## Background

Coronavirus disease 2019 (COVID-19) has been shown to cause serious health problems. The most devastating complication of this disorder is the Acute Respiratory Distress syndrome (ARDS) [1] which is thought to be caused by a mixture of mechanisms among them are cytokine storm [2], abnormal activity of the renin-angiotensin apparatus [3], neutrophil stimulation [4] and enhanced coagulation [5]. This COVID-19 complication has been shown to be provoked by vitamin D deficiency and attenuated by induction of the vitamin D receptor (VDR) [6]. This observation is supported by the presence of VDR on immune cells, the regulatory role of the active vitamin D hormone on expression of the majority of cytokines and the role of this hormone on activation of immune defense responses while attenuation of the acquired immune responses [7–9]. Antimicrobial function of vitamin D is exerted through a cascade of events. Macrophages respond to microbial infection through pattern recognition receptors. Induction of these responses leads to activation of transcription of 1α-hydroxylase (CYP27B1) and VDR [10]. The impact of vitamin D on regulation of immune responses is modulated by availability of 25-hydroxyvitamin D, activation of CYP27B1 by the attacking pathogenic organisms and induction of 1,25-dihydroxyvitamin D in cellular compartments of the immune system [10, 11]. Moreover, expression of VDR has been shown to be affected by several mechanisms among them are long non-coding RNAs (lncRNAs) [12]. We have previously assessed expression of VDR-associated lncRNAs namely *SNHG16*, *SNHG6*, *LINC00346* and *LINC00511* in a number of disorders including epilepsy [13], lung cancer [14] and breast cancer [15]. In the current project, we have appraised expression levels of *VDR*, *CYP27B1* and mentioned lncRNAs in the circulation of COVID-19 patients versus healthy subjects to unravel the role of these transcripts in the pathogenic processes during the course of COVID-19.

## Methods

### Enrolled individuals

This study is a pilot study to measure expression levels of *VDR* and related lncRNAs in COVID-19 cases and healthy controls. The current investigation was conducted on patients hospitalized to Nikan Hospital, Tehran, during March 2020 until April 2020. Patients have clinical manifestations of COVID-19 and the disorder was confirmed by a positive nasopharyngeal swab specimen. Patients were diagnosed to have moderate, severe and very severe disease courses and were assigned hospital treatment [16]. Control specimens were got from healthy persons without no clinical signs or recent exposure to patients with COVID-19. The study protocol was approved by ethical committee of Shahid Beheshti University of Medical Sciences. Informed consent was obtained from all patients. Paraclinical data was obtained from all hospitalized patients. Hematological tests were performed in Beckman Coulter MAXM AL Hematology Flow Cytometry System. C-reactive protein (CRP) was measured by latex-enhanced nephelometry.

### Expression assays

First, 4 ml of peripheral blood were collected from all hospitalized patients and healthy persons. Subsequently, total RNA was isolated from these specimens using the GeneAll Kit (Seoul, South Korea). Then, total RNA was transformed to cDNA using the OneStep RT-PCR Series Kit (BioFact™, Seoul, South Korea). Transcript quantities of VDR-associated genes were measured in specimens gathered from COVID-19 patients and healthy subjects using the RealQ Plus 2x Master Mix (Amplicon, Denmark). Primers Characteristics have been reported in our previous studies [13, 15].

### Data analysis

R programming language was used for data analysis. Transcript quantities of VDR-associated genes were quantified from Ct values, considering *B2M* as the reference gene. The obtained values were log2 transformed and used for next steps. Expression levels of genes were compared between COVID-19 patients and healthy subjects and ICU-hospitalized and non-ICU hospitalized patients. The significance of difference in mean values of gene expression between two subgroups was computed using the t-test. Correlations between expression levels of genes were appraised via calculation of Spearman correlation coefficients. ROC curves were depicted using Bayesian Generalized Linear Model, Generalized Linear Model (GLM), and Linear Discriminant Analysis with 10-fold cross validation. Application of the GLM resulted in the most appropriate estimates. Youden’s J parameter was measured to find the optimum threshold. *P* value < 0.05 was considered as significant.

## Results

Totally, 91 COVID-19 patients (38 females, 53 males) and 91 healthy subjects (39 females, 52 males) were included in the study. The mean age (±standard deviation) of the COVID-19 patients was 57.18 (±16.89) years. Thirty seven patients (40.6%) were hospitalized in the ICU. Table 1 displays the paraclinical variables obtained from medical records of the COVID-19 patients.

**Table 1:** Paraclinical data of the COVID-19 patients

Variable	Mean	Standard deviation
White Blood Cells (10^9^/L)	8.119	8.482
Red Blood Cells (10^12^/L)	4.681	0.768
Hemoglobin (g/dL)	12.704	2.182
Hematocrit (%)	39.267	6.595
MCV (fl)	83.985	5.702
MCH (pg)	27.154	2.330
MCHC (g/dL)	32.348	1.3720
Platelet count (10^9^/L)	210.354	95.216
Lymphocyte (%)	21.043	11.323
Neutrophil (%)	69.098	13.087
ESR (mm/hr)	44.131	32.701
CRP (mg/dL)	73.256	69.540

### Expression assays

**Fig. 1:**
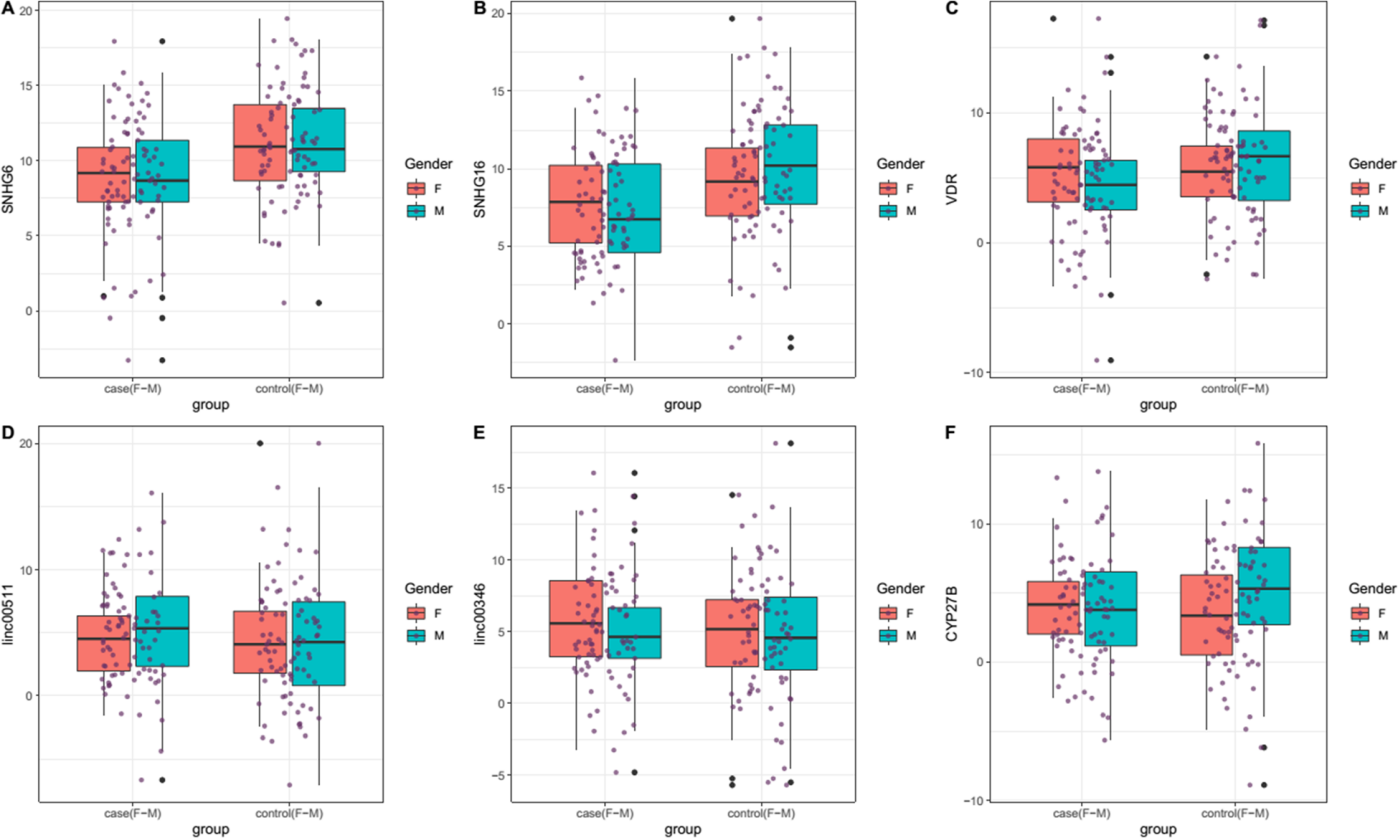
Relative expressions of *VDR*, *CYP27B* and *SNHG6*, *SNHG16*, *Linc00511* and *Linc00346* lncRNAs in total COVID-19 patients compared with controls

**Fig. 2:**
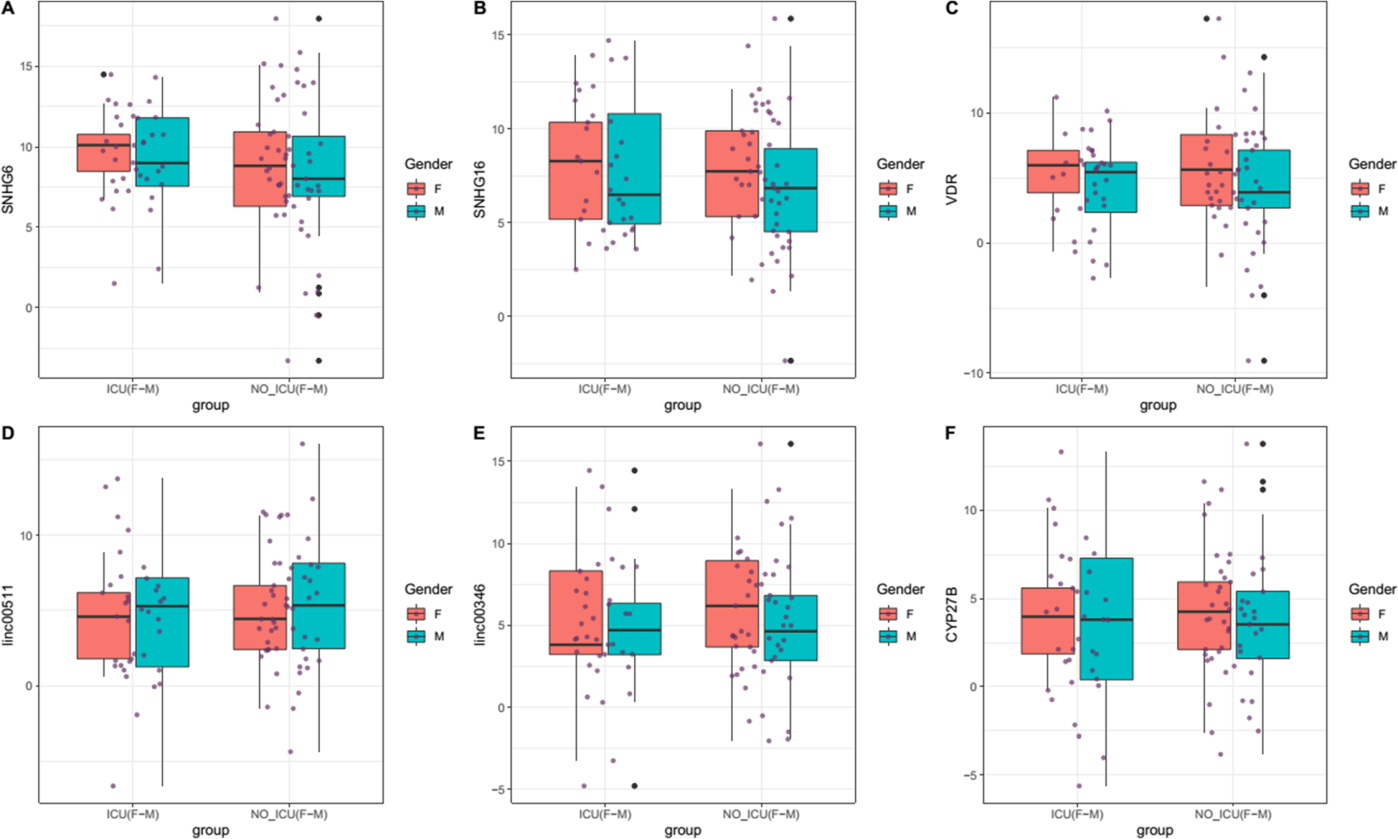
Relative expressions of *VDR*, *CYP27B* and *SNHG6*, *SNHG16*, *Linc00511* and *Linc00346* lncRNAs in ICU-hospitalized patients compared with non-ICU patients

Figures 1 and 2 depict relative expressions of *VDR*, *CYP27B* and *SNHG6*, *SNHG16*, *Linc00511* and *Linc00346* lncRNAs in total COVID-19 patients compared with healthy persons, and in ICU-hospitalized patients compared with non-ICU patients, respectively.

Expression of *SNHG6* was remarkably lower in COVID-19 patients compared with controls (Ratio of mean expression (RME) = 0.22, *P* value = 7.04E-05) and in both female and male affected individuals compared with sex-matched controls (RME = 0.32, P value = 0.04 and RME = 0.16, *P* value = 0.000679683, respectively). However, its expression was statistically similar between ICU-hospitalized and non-ICU hospitalized patients. Similarly, expression of *SNHG16* was lower in in COVID-19 patients compared with controls (RME = 0.20, *P* value = 5.94E-05) and in both female and male affected individuals compared with sex-matched healthy subjects (RME = 0.32, *P* value = 0.04 and RME = 0.14, P value = 0.000496435, respectively) with no significant difference among ICU-hospitalized and non-ICU hospitalized patients. Expression of *VDR* was lower in COVID-19 patients compared with controls (RME = 0.42, *P* value = 0.04) and in male patients compared with normal males (RME = 0.27, P value = 0.02). However, expression of VDR was statistically similar between female patients and normal females and between ICU-hospitalized and non-ICU hospitalized patients. Expression levels *CYP27B*, *Linc00511* and *Linc00346* were not different between COVID-19 patients and healthy subjects or between their subgroups (Table 2).

**Table 2:** Detailed statistics of expression of *VDR*, *CYP27B* and *SNHG6*, *SNHG16*, *Linc00511* and *Linc00346* lncRNAs in COVID-19 patients and controls (RME: Ratio of mean expression)

		***SNHG6***					***SNHG16***					***VDR***					***Linc00511***					***Linc00346***					***CYP27B***				
Number of Samples		SE	RME	P Value	95% CI		SE	RME	P Value	95% CI		SE	RME	P Value	95% CI		SE	RME	P Value	95% CI		SE	RME	P Value	95% CI		SE	RME	P Value	95% CI	
**Cases/ controls**																															
**Total**	**91/91**	0.54	0.22	0.00	−3.29	−1.14	0.56	0.20	0.00	−3.40	− 1.20	0.61	0.42	0.04	−2.44	−0.05	0.64	1.55	0.33	−0.64	1.90	0.62	1.34	0.50	−0.80	1.64	0.61	0.71	0.43	−1.69	0.72
**F**	**38/39**	0.78	0.32	0.04	−3.21	−0.09	0.79	0.32	0.04	−3.22	−0.05	0.89	0.78	0.68	−2.14	1.41	0.86	1.01	0.99	−1.71	1.74	0.98	1.98	0.32	−0.96	2.93	0.79	1.39	0.54	−1.09	2.04
**M**	**53/52**	0.75	0.16	0.00	−4.11	−1.14	0.78	0.14	0.00	−4.33	−1.25	0.82	0.27	0.02	−3.51	−0.25	0.92	2.11	0.25	−0.75	2.90	0.81	1.01	0.99	−1.59	1.61	0.88	0.43	0.17	−2.96	0.53
**ICU/ Non-ICU**																															
**Total**	**37/54**	0.75	1.74	0.29	−0.69	2.30	0.75	1.49	0.44	−0.91	2.06	0.84	0.90	0.85	−1.82	1.51	0.87	0.60	0.40	−2.47	0.99	0.84	0.73	0.59	−2.12	1.21	0.85	0.79	0.68	−2.03	1.34
**F**	**13/25**	0.97	2.18	0.26	−0.85	3.10	1.09	1.11	0.90	−2.12	2.41	1.29	0.90	0.91	−2.78	2.48	1.01	0.62	0.51	−2.74	1.38	1.46	0.59	0.61	−3.77	2.25	1.03	0.92	0.91	−2.22	1.99
**M**	**24/29**	1.10	1.64	0.52	−1.49	2.92	1.04	1.93	0.37	−1.14	3.04	1.12	1.05	0.95	−2.18	2.33	1.27	0.52	0.46	−3.50	1.61	1.03	0.90	0.88	−2.23	1.92	1.23	0.71	0.69	−2.98	1.98

**Fig. 3:**
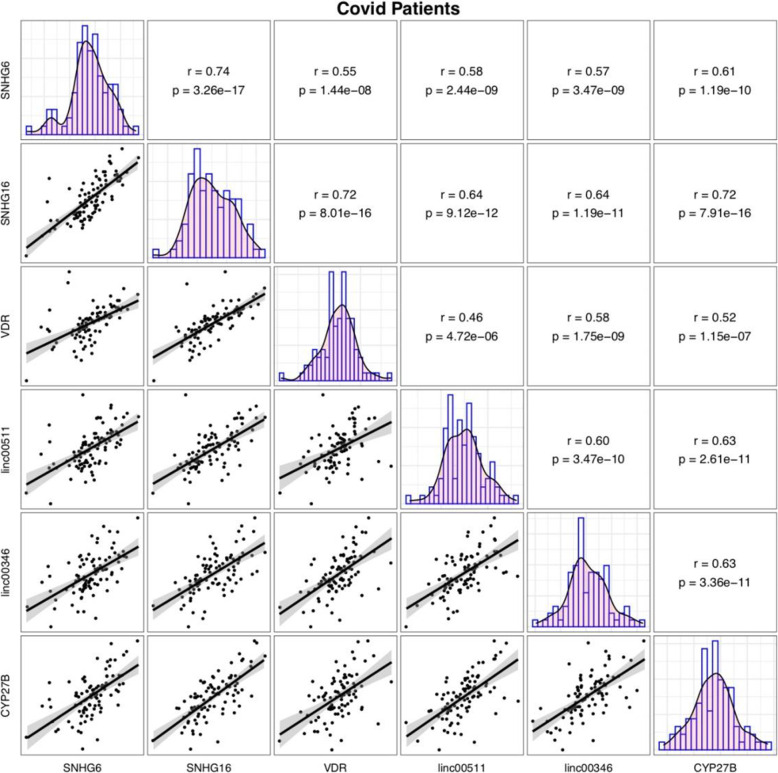
Correlation between expressions of *VDR*, *CYP27B* and *SNHG6*, *SNHG16*, *Linc00511* and *Linc00346* in COVID-19 patients

**Fig. 4:**
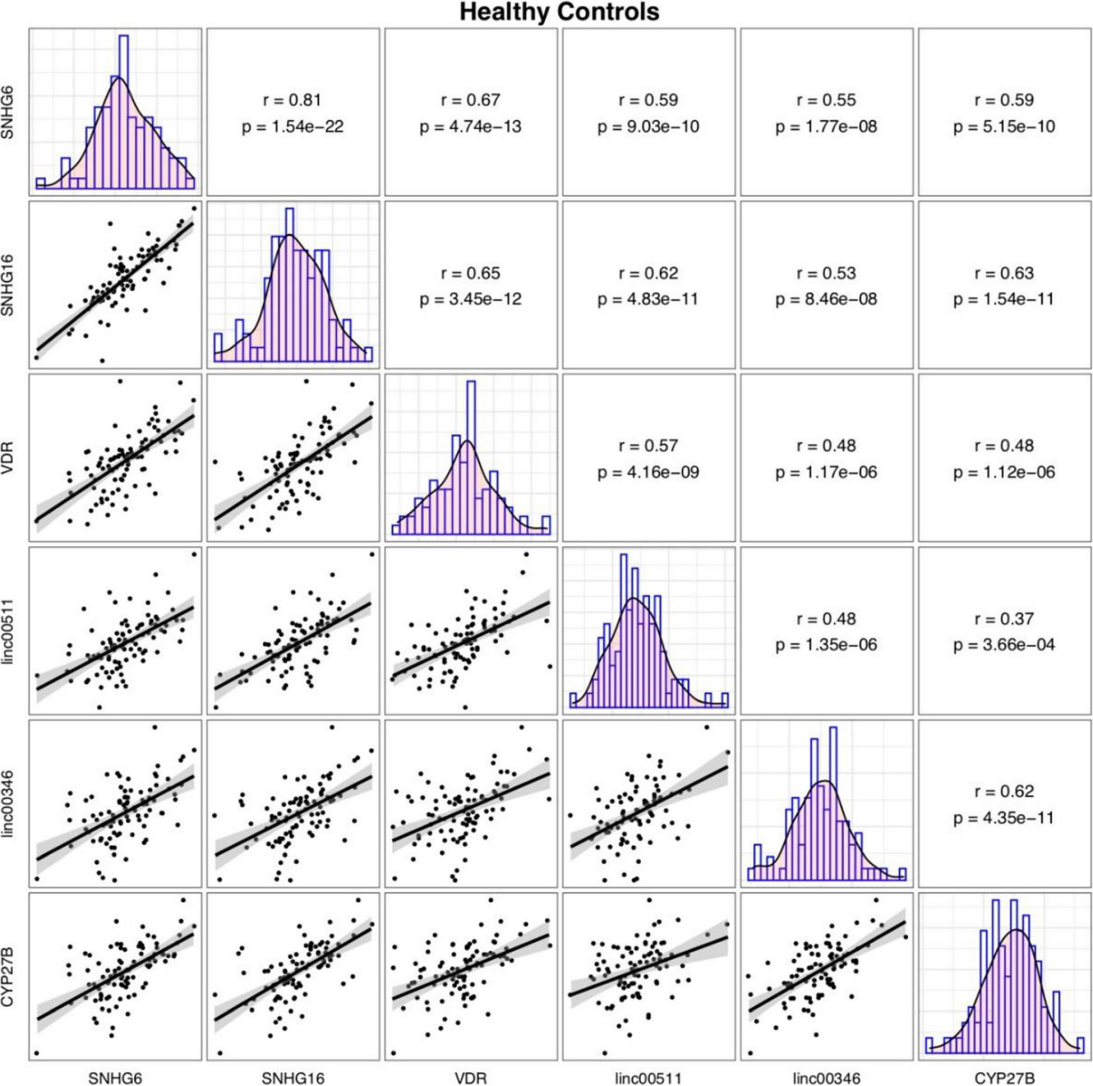
Correlation between expressions of *VDR*, *CYP27B* and *SNHG6*, *SNHG16*, *Linc00511* and *Linc00346* in controls

Significant correlations have been displayed between expression levels of *VDR*, *CYP27B* and *SNHG6*, *SNHG16*, *Linc00511* and *Linc00346* lncRNAs both among COVID-19 patients and among healthy controls with the most significant ones being *SNHG6* and *SNHG16* (r = 0.74, *P* value = 3.26e-17 and r = 0.81, *P* = 1.54e-22, respectively) (Figs. 3 and 4).

**Fig. 5:**
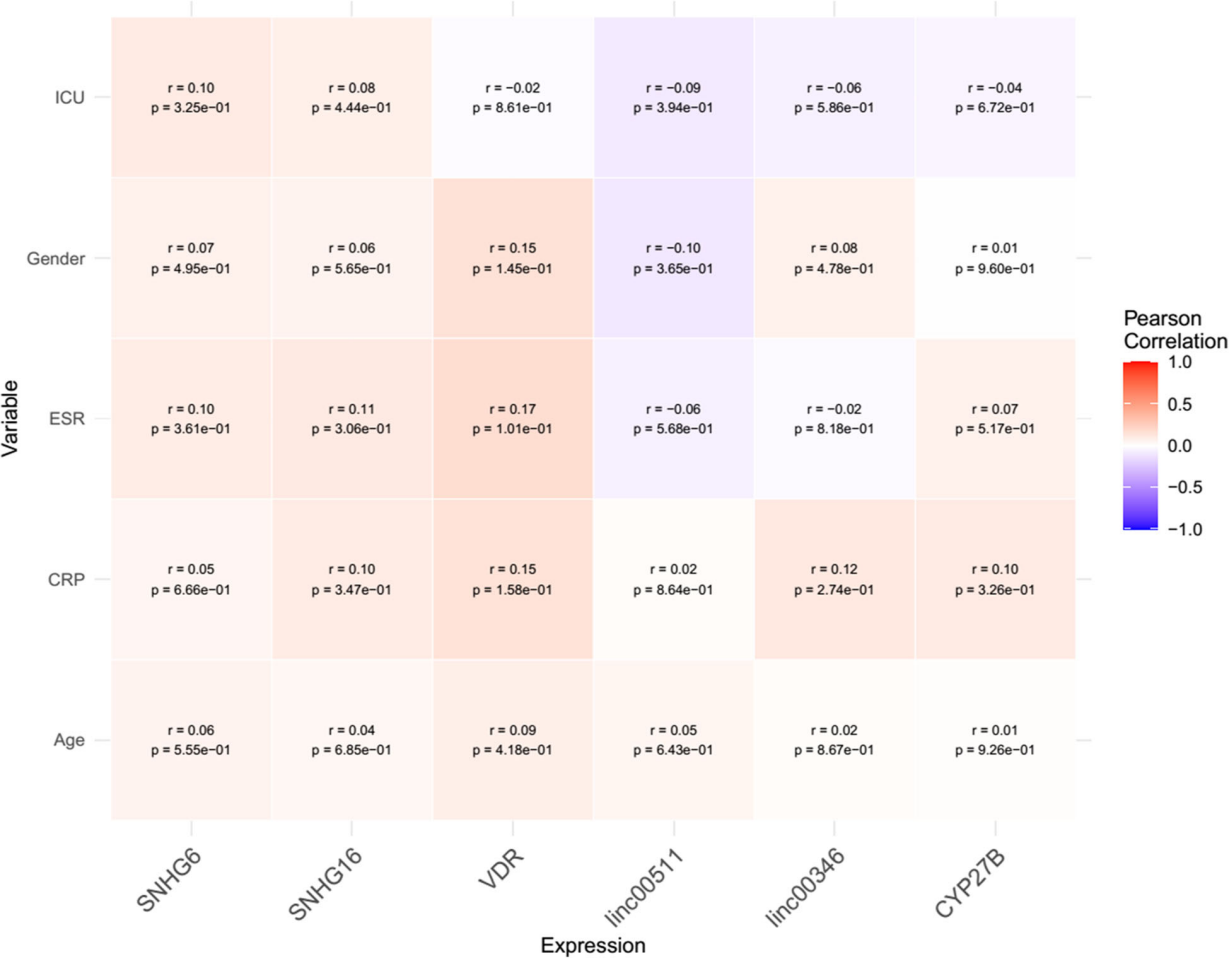
Association between expression levels of *VDR*, *CYP27B* and *SNHG6*, *SNHG16*, *Linc00511* and *Linc00346* and clinical/demographic data

Next, we appraised correlation between expression levels of mentioned genes and patients’ gender and age as well as some paraclinical parameters demonstrating no robust correlations (Fig. 5).

**Fig. 6:**
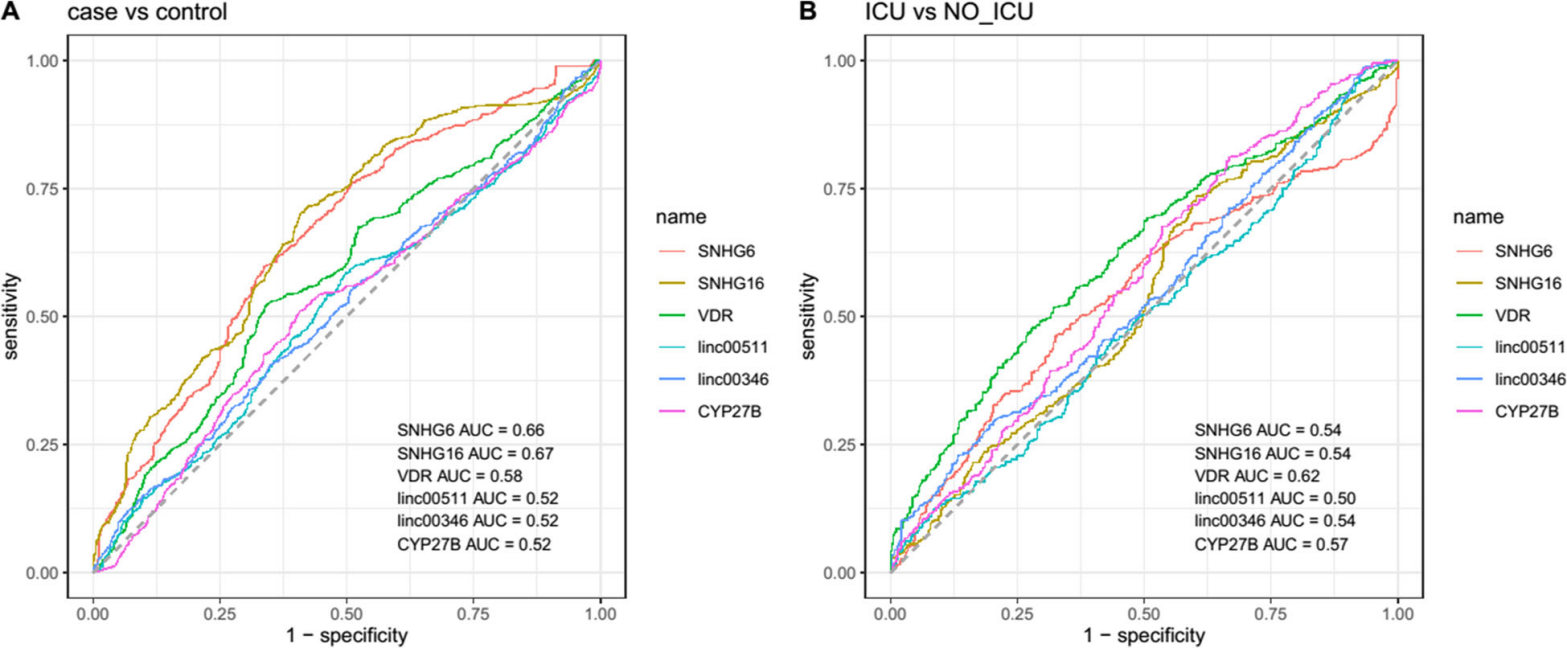
ROC curves demonstrating the diagnostic power of *VDR*, *CYP27B* and *SNHG6*, *SNHG16*, *Linc00511* and *Linc00346* in distinguishing COVID-19 patients from healthy controls (A) and in distinguishing ICU-hospitalized patients from the other group of patients (B)

Finally, the diagnostic power of *VDR*, *CYP27B* and *SNHG6*, *SNHG16*, *Linc00511* and *Linc00346* has been appraised in distinguishing COVID-19 patients from healthy controls and in distinguishing ICU-hospitalized patients from the other group of patients (Fig. 6).

While none of the individual genes could appropriately distinguish COVID-19 patients from healthy controls or ICU-hospitalized patients from non-ICU-hospitalized one, combination of transcript levels of *VDR*, *CYP27B* and *SNHG6*, *SNHG16*, *Linc00511* and *Linc00346* could differentiate patients from controls with AUC = 0.76, sensitivity = 0.62 and specificity = 0.81. However, this type of analysis revealed no suitable diagnostic power for distinguishing ICU-hospitalized patients from non-ICU-hospitalized one (Table 3).

**Table 3:** Detailed parameters of ROC curve assessment for appraisal of the diagnostic power of genes in distinguishing COVID-19 patients from healthy controls and in distinguishing ICU-hospitalized patients from the non-ICU patients (Se: sensitivity, Sp: specifciicty)

		***SNHG6***			***SNHG16***			***VDR***			***Linc00511***			***Linc00346***			***CYP27B***			All		
Number of Samples		AUC	Se	Sp	AUC	Se	Sp	AUC	Se	Sp	AUC	Se	Sp	AUC	Se	Sp	AUC	Se	Sp	AUC	Se	Sp
**Total patients/ controls**																						
**Total**	**91/91**	0.66	0.60	0.66	0.67	0.70	0.59	0.58	0.52	0.66	0.52	0.58	0.50	0.52	0.41	0.65	0.52	0.51	0.59	0.76	0.62	0.81
**ICU/Non_ICU**																						
**Total**	**37/54**	0.54	0.46	0.67	0.54	0.74	0.40	0.62	0.48	0.72	0.50	0.97	0.08	0.54	0.23	0.87	0.57	0.81	0.33	0.53	0.25	0.84

## Discussion

Vitamin D deficiency and defects in activation of VDR have been found to intensify respiratory syndrome in COVID-19 through inducing a wounding response in stellate cells of the respiratory system [17]. Moreover, vitamin D levels have been found to be associated with the number of COVID-19 cases/million as well as number of deaths from COVID-19/ million [18]. Based on the presence of extensive evidence regarding the role of VDR signaling in the pathogenic course of COVID-19 and its devastating complication i.e. ARDS, we have appraised expression levels of *VDR*, *CYP27B1* and some VDR-associated lncRNAs in the peripheral blood of COVID-19 patients versus healthy subjects. We reported lower expressions of *SNHG6* and *SNHG16* in COVID-19 patients of both sexes compared with sex-matched controls. In addition, expression of *VDR* was lower in COVID-19 patients compared with controls and in male patients compared with male controls. Conversely, expression of *VDR* was statistically similar between female patients and normal females. Expression of none of genes was different between ICU-hospitalized and non-ICU hospitalized patients. *SNHG6* has been shown to activate TGF-β/Smad signaling pathway [19]. The interaction between coronaviruses and this pathway is complicated. For instance, SARS-associated coronavirus (SARS-CoV) nucleocapsid protein has been shown to enhance TGF-β-associated expression of PAI-1 but decreasing Smad3/Smad4-asociated apoptosis of lung epithelial cells [20]. On the other hand, SARS-CoV-2 infection has been shown to increase TGF-β production [21]. Moreover, in severe COVID-19, SARS-CoV-2 has been found to induce TGF-β-dominated chronic immune responses that do not target SARS-CoV-2 antigens [22]. Several viruses alter TGF-β signaling to inhibit cell apoptosis and enhance proliferation of fibroblasts and differentiation of myofibroblasts [23]. Over-production of TGF- β has been reported in acute-phase SARS and COVID-19 in relation with the development of lung fibrosis. Moreover, this phenomenon has been detected in autopsy samples and a considerable percentage of SARS, MERS and COVID-19 survivors [17]. Therefore, up-regulation of TGF-β in COVID-19 patients might contribute in the lung fibrosis [21]. The observed down-regulation of *SNHG6* in the peripheral blood of COVID-19 patients might be due to a regulatory feedback loop between this lncRNA and TGF-β.

*SNHG16* has been shown to activate TGF-β1/SMAD5 pathway via miR-16–5p/SMAD5-regulatory cascade, therefore activating CD73 expression in γδ1 T cells [24]. Down-regulation of *SNHG16* in COVID-19 patients might result in decreased proportion of γδ1 T cells, thus activation of pro-inflammatory cascades and pulmonary fibrosis, since some subsets of γδ T cells regulate immunosuppressive functions and induce immune tolerance in certain contexts [25].

It is not clear whether the observed decreased level of *VDR* in COVID-19 is the cause or effects of COVID-19. However, previous studies have indicated that certain polymorphisms in the *VDR* gene might have adverse impact on the outcome of patients with COVID-19 [26, 27]. Therefore, we suggest simultaneous assessment of functional polymorphisms within this gene and its expression in larger populations of COVID-19 patients to distinguish the possible correlations. Moreover, VDR has been found to transcriptionally silence TGF-β signaling via genomic competition with Smad3 recruitment on pro-fibrotic and pro-inflammatory genes [28]. Therefore, the observed down-regulation of *VDR* in COVID-19 cases might result in up-regulation of TGF-β signaling and related pathologic events.

Expression levels *CYP27B*, *Linc00511* and *Linc00346* were not different between COVID-19 patients and healthy subjects or between their subgroups, implying their independence from COVID-19 infection or disease course.

Significant correlations have been displayed between expression levels of *VDR*, *CYP27B* and *SNHG6*, *SNHG16*, *Linc00511* and *Linc00346* lncRNAs both among COVID-19 patients and among normal controls with the most significant ones being *SNHG6* and *SNHG16*. These two lncRNAs have also been shown to correlate with each other in other disease contexts [13], implying their functional interactions in different situations. Expression of these genes were not correlated with paraclinical data indicating the independence of these transcripts from these parameters particularly inflammation markers.

Consistent with the similar levels of expressions of genes between ICU-hospitalized patients and other patients, these genes could not distinguish these two subgroups of patients. However, combination of transcript levels of *VDR*, *CYP27B* and *SNHG6*, *SNHG16*, *Linc00511* and *Linc00346* could differentiate patients from controls with moderate power.

Taken together, the current data potentiate *SNHG6*, *SNHG16* and *VDR* as possible contributors in the COVID-19 infection but not in the severity of ARDS. Future studies should attempt to uncover the fundamental mechanism of this possible contribution and unravel the molecular mediators in this pathway. However, we do not have the data of 25(OH) D levels of patients and controls. We state this point as a limitation of our study.

## Data Availability

The analysed data sets generated during the study are available from the corresponding author on reasonable request.

## Abbreviations

COVID-19: Coronavirus disease 2019
ARDS: Acute Respiratory Distress syndrome
VDR: Vitamin D receptor
lncRNAs: long non-coding RNAs
CRP: C-reactive protein
SARS-CoV: SARS-associated coronavirus
